# Bioglass/ceria nanoparticle hybrids for the treatment of seroma: a comparative long-term study in rats

**DOI:** 10.3389/fbioe.2024.1363126

**Published:** 2024-03-12

**Authors:** Michael-Alexander Pais, Athanasios Papanikolaou, Isabel Arenas Hoyos, Robert Nißler, Simone De Brot, Alexander Gogos, Robert Rieben, Mihai A. Constantinescu, Martin T. Matter, Inge K. Herrmann, Ioana Lese

**Affiliations:** ^1^ Department of Plastic and Hand Surgery, Inselspital, University Hospital Bern, Bern, Switzerland; ^2^ Department for BioMedical Research, University of Bern, Bern, Switzerland; ^3^ Department of Materials Meet Life, Swiss Federal Laboratories for Materials Science and Technology (Empa), StGallen, Switzerland; ^4^ Department of Mechanical and Process Engineering, ETH Zurich, Zurich, Switzerland; ^5^ Ingenuity Lab, University Hospital Balgrist and University of Zurich, Zurich, Switzerland; ^6^ COMPATH, Institute of Animal Pathology, University of Bern, Bern, Switzerland

**Keywords:** seroma formation, bioglass/ceria nanoparticle hybrids, animal rat model, anti-inflammatory response of nanoparticles, adhesive properties of nanoparticles

## Abstract

**Background:** Seroma formation is a common postoperative complication. Fibrin-based glues are typically employed in an attempt to seal the cavity. Recently, the first nanoparticle (NP)-based treatment approaches have emerged. Nanoparticle dispersions can be used as tissue glues, capitalizing on a phenomenon known as ‘nanobridging’. In this process, macromolecules such as proteins physically adsorb onto the NP surface, leading to macroscopic adhesion. Although significant early seroma reduction has been shown, little is known about long-term efficacy of NPs. The aim of this study was to assess the long-term effects of NPs in reducing seroma formation, and to understand their underlying mechanism.

**Methods:** Seroma was surgically induced bilaterally in 20 Lewis rats. On postoperative day (POD) 7, seromas were aspirated on both sides. In 10 rats, one side was treated with NPs, while the contralateral side received only NP carrier solution. In the other 10 rats, one side was treated with fibrin glue, while the other was left untreated. Seroma fluid, blood and tissue samples were obtained at defined time points. Biochemical, histopathological and immunohistochemical assessments were made.

**Results:** NP-treated sides showed no macroscopically visible seroma formation after application on POD 7, in stark contrast to the fibrin-treated sides, where 60% of the rats had seromas on POD 14, and 50% on POD 21. At the endpoint (POD 42), sides treated with nanoparticles (NPs) exhibited significant macroscopic differences compared to other groups, including the absence of a cavity, and increased fibrous adhesions. Histologically, there were more macrophage groupings and collagen type 1 (COL1) deposits in the superficial capsule on NP-treated sides.

**Conclusion:** NPs not only significantly reduced early manifestations of seroma and demonstrated an anti-inflammatory response, but they also led to increased adhesion formation over the long term, suggesting a decreased risk of seroma recurrence. These findings highlight both the adhesive properties of NPs and their potential for clinical therapy.

## 1 Introduction

A seroma is an encapsulated accumulation of serous fluid which may occur after certain surgeries, with an incidence of up to 85% of patients (Kumar et al., 1995). The most common surgeries where seroma formation can be expected postoperatively are abdominoplasty (Rousseau et al., 2011; Marsh et al., 2015; Anker et al., 2021), mastectomy with or without axillary dissection (Kumar et al., 1995) and the donor site after harvesting the latissimus dorsi muscle for reconstructive purposes (Llewellyn-Bennett et al., 2012). Seroma is associated with a multitude of adverse effects, such as, increased risk of infection, wound dehiscence, prolonged drainage, recurrent aspirations, multiple hospital admissions, recurrent surgeries, and frequent follow-ups which makes the current therapeutic management of seroma troublesome (Kuroi et al., 2005; Anker et al., 2021; Papanikolaou et al., 2022). Treatment options in seroma management include various non-surgical and surgical options, from application of sclerosing agents into the seroma, serial aspirations, prolonged drainage, to surgical marsupialization or debridement of the seroma capsule (Beidas and Gusenoff, 2019; Papanikolaou et al., 2022). There have also been studies looking at prevention strategies to avoid the formation of seroma, such as drainage placement during the initial surgery (Unalp and Onal, 2007; Papanikolaou et al., 2022; Shipkov and Uchikov, 2023), compression garments (Chen et al., 1998; Brown et al., 2023; Shipkov and Uchikov, 2023), and even treatment with fibrin glue (Llewellyn-Bennett et al., 2012; Pollock and Pollock, 2012; Azoury et al., 2015). But yet standard of care protocols and management recommendations in a clinical setting are missing.

Although seroma pathogenesis remains unclear, it includes both mechanical and inflammatory elements of surgical trauma (Watt-Boolsen et al., 1989; McCaul et al., 2000). Surgical injury triggers an immunomodulatory process, similar to the pathophysiology of seroma formation after peritoneal trauma (Maciver et al., 2011; Fang et al., 2018; Anker et al., 2021). The current thinking for pathways responsible for seroma formation also includes the plasmin system and components of the coagulation cascade (Oertli et al., 1994; Bonnema et al., 1999). In the literature, a lack of fibrinogen was described postoperatively, with the attempt of then supplementing it with coagulation factors to promote coagulation (Kulber et al., 1997; Llewellyn-Bennett et al., 2012). For example, supplementation with fibrin sealant has previously shown efficacy in a rat model (Kulber et al., 1997), but it failed to show any benefit for minimizing seromas in a randomized controlled trial in the clinical setting (Llewellyn-Bennett et al., 2012). Commonly, fibrin sealant is used in combination with tensile-reducing quilting sutures (Sajid et al., 2011; Papanikolaou et al., 2022), but its limitations are the expenses of fibrin glue and the added surgical time matter, on the other hand (Pollock and Pollock, 2012; Azoury et al., 2015). Another approach was based on the idea of preventing fibrinolysis with the consequence of degradation of already formed fibrin complexes, using tranexamic acid, an antifibrinolytic agent (Oertli et al., 1994). This randomized controlled trial failed though to show any statistically significant results (Oertli et al., 1994). A variety of tissue adhesives and substances have been used to reduce postoperative seroma formation (Chung et al., 2006; Gilbert et al., 2013; Qvamme et al., 2015), but a satisfactory remedy has yet to be determined. With the goal of closing any remaining dead space and preventing further drainage, a treatment should bond dead-space walls and maintain integrity despite shear forces.

Nanotechnology has evolved tremendously in the last years (Oertli et al., 1994; Bonnema et al., 1999; Maciver et al., 2011; Fang et al., 2018). Rose et al. (Rose et al., 2014) first described protein adhesive properties of inorganic silica and iron oxide nanoparticles (NPs) leading to macroscopic adhesions of liver tissue. Integrating additional bioactivity, hybrid Ceria (CeO2)/bioglass (BG) tissue glues have been developed, with importance especially in the field of bone tissue engineering and soft tissue regeneration (Hench et al., 2004; Boccaccini and Blaker, 2005; Yu et al., 2016). Further on, these particles have been enhanced with elements exhibiting potent bioactivity, such as strontium (Sr) and zinc (Zn) (Nielsen, 1990; Beattie and Avenell, 1992). Both elements are known to exert angiogenic, and Zn also anti-inflammatory properties (Lang et al., 2007; Hoppe et al., 2011). Within a previous study of our group (Lese et al., 2018), Zinc doped Strontium substituted bioglass/ceria NPs were shown to increase skin flap survival in a rat model through neo-angiogenic and anti-inflammatory mechanisms, and to reduce seroma formation without any detectable systemic adverse effects in a short-term setting of 2 weeks (Lese et al., 2021). However, for clinical translation, the mechanistic understanding and long-term fate of NP-based tissue adhesives is imperative. Thus, in this study, we comprehensively investigated the benefits and drawbacks of NP-based tissue glues in clinically relevant seroma scenarios. The seroma model is based on various rat models for seroma formation (Harada et al., 1992; Kulber et al., 1997; Chung et al., 2006; Choi et al., 2012; Hurwitz et al., 2015; Lese et al., 2021). Based on the Kulber et al. animal model, and on the model used in our previous short-term study, we opted for the same bilateral seroma rat model, where we successfully demonstrated surgically induced seroma formation (Kulber et al., 1997; Lese et al., 2021). We demonstrate that the use of nanoparticles effectively diminishes early signs of seroma and exhibit anti-inflammatory effects. Moreover, they promote long-term adhesion formation, implying a reduced likelihood of seroma recurrence. These outcomes underscore the dual benefits of NP-based seroma treatments: their adhesive capabilities and their promising therapeutic value in clinical settings.

## 2 Materials and methods

### 2.1 Nanoparticle synthesis

Zinc-doped, strontium-substituted, bioglass/ceria NPs were produced by flame spray pyrolysis, according to previously described methods in the literature (Matter et al., 2017; Lese et al., 2018; Lese et al., 2021). First, calcium 2-ethylhexanoate, sodium 2-ethylhexanoate, tributyl phosphate, hexamethyldisiloxane, strontium acetylacetonate hydrate, Ce-2-ethylhexanoate, and zinc acetylacetonate were diluted in tetrahydrofuran (THF), and then sprayed through a nozzle. As a next step, after ignition by a flame of the droplets of the initial mixture, a nucleation process took place, followed by collection of the created NPs in form of a powder of fine dispersed particles. NPs were then dispersed in the given concentration in a modified Ringer’s lactate buffer, containing citric acid and sodium citrate at 7.5 mM each (Starsich et al., 2017; Matter et al., 2019). NPs were stored in Eppendorf tubes at room temperature before dispersion. The nanomaterials, prepared as such, are readily available for immediate use in biomedical applications, as previously mentioned (Lese et al., 2018; Matter et al., 2020).

### 2.2 Nanoparticle characterization

As synthesized NPs were analysed via X-ray diffraction (XRD) using a Bruker D2 second Gen Phaser (40 kV, 40 mA, Cu Kα radiation at 2θ = 10°–80° with a step size of 0.03°). Phase composition was determined using the Diffrac Eva (V3.1) software (Matter et al., 2019). The surface area was determined according to the Brunauer−Emmett− Teller (BET) method at 77 K (Micromeritics, Tristar II Plus) (Starsich et al., 2017). Elemental composition was analysed by following the optimized NP digestion protocol as outlined in section 2.5, via ICP-OES (Agilent, Santa Clara, CA) (Oertli et al., 1994; Kulber et al., 1997).

### 2.3 Surgical model of seroma induction

A bilateral seroma induction surgery was performed as described previously in our rat model (Lese et al., 2021): an incision was placed in the posterior axillary line, the cutaneous maximus and latissimus dorsi muscles were excised, and an axillary lymphadenectomy was performed. Additionally, the under surface of the skin flap was also scraped with a scalpel. Skin closure was performed with Vicryl 4–0 sutures (Coated VICRYL™ (polyglactin 910), ^©^ Ethicon US, LLC).

All surgical and interventional procedures in rats were performed using balanced anaesthesia: fentanyl 0.0005 mg/kg, medetomidine 0.15 mg/kg and midazolam 2 mg/kg, administered subcutaneously (s.c.). After administering this anaesthetic cocktail, oxygen (O_2_) was administered until loss of consciousness. During surgery, 100% O_2_ was used. In cases of prolonged anaesthesia, inhalational maintenance anaesthesia was administers using 1.0%–2.0% Isoflurane. Rats were maintained at normal body temperature using thermal pads, and ophthalmic ointment was applied to the eyes. Continuous monitoring (respiratory rate, temperature) was provided until awake. At the end of surgery, anaesthesia was reversed with a cocktail containing buprenorphine 0.05 mg/kg, atipamezole 0.75 mg/kg and flumazenil 0.2 mg/kg s. c. For continuous analgesia meloxicam 1 mg/kg was administered postoperatively. During recovery from anaesthesia, rats were kept warm using a heating pad for at least 1 h. Analgesia was continued for 48 h postoperatively in drinking water (360 mL) containing both buprenorphine 0.3 mg/mL and 10 mL 5% glucose, and with meloxicam 1 mg/kg for 4 days. Rescue analgesia with buprenorphine was given when rats showed signs of additional pain, according to score-sheet assessments (Lese et al., 2021).

Soft critical-care feed (EmerAid Omnivore, EmerAid, LLC, Cornell, IL, USA) was provided to support feeding and recovery for the first five post-operative days.

For weekly blood sampling and seroma aspiration, isoflurane (5% with 100% O_2_, 1 L/min) was used for initial anaesthesia in an induction chamber. Maintenance anaesthesia was provided using 2%–2.5% isoflurane with 0.6% L/min O2.

For euthanasia rats underwent terminal anesthesia using 150 mg/kg pentobarbital administered intraperitoneally (i.p.) (Esconarkon, 300 mg/mL; dilution 1:10; Streuli Pharma, Uznach, Switzerland).

This study was conducted according to the ARRIVE guidelines (Percie du Sert et al., 2020), and was approved by the Cantonal Animal Ethics Committee for Animal Experimentation, Bern, Switzerland (approval number BE 110/2020).

### 2.4 Treatment group allocation and experimental design setup

**FIGURE 1:**
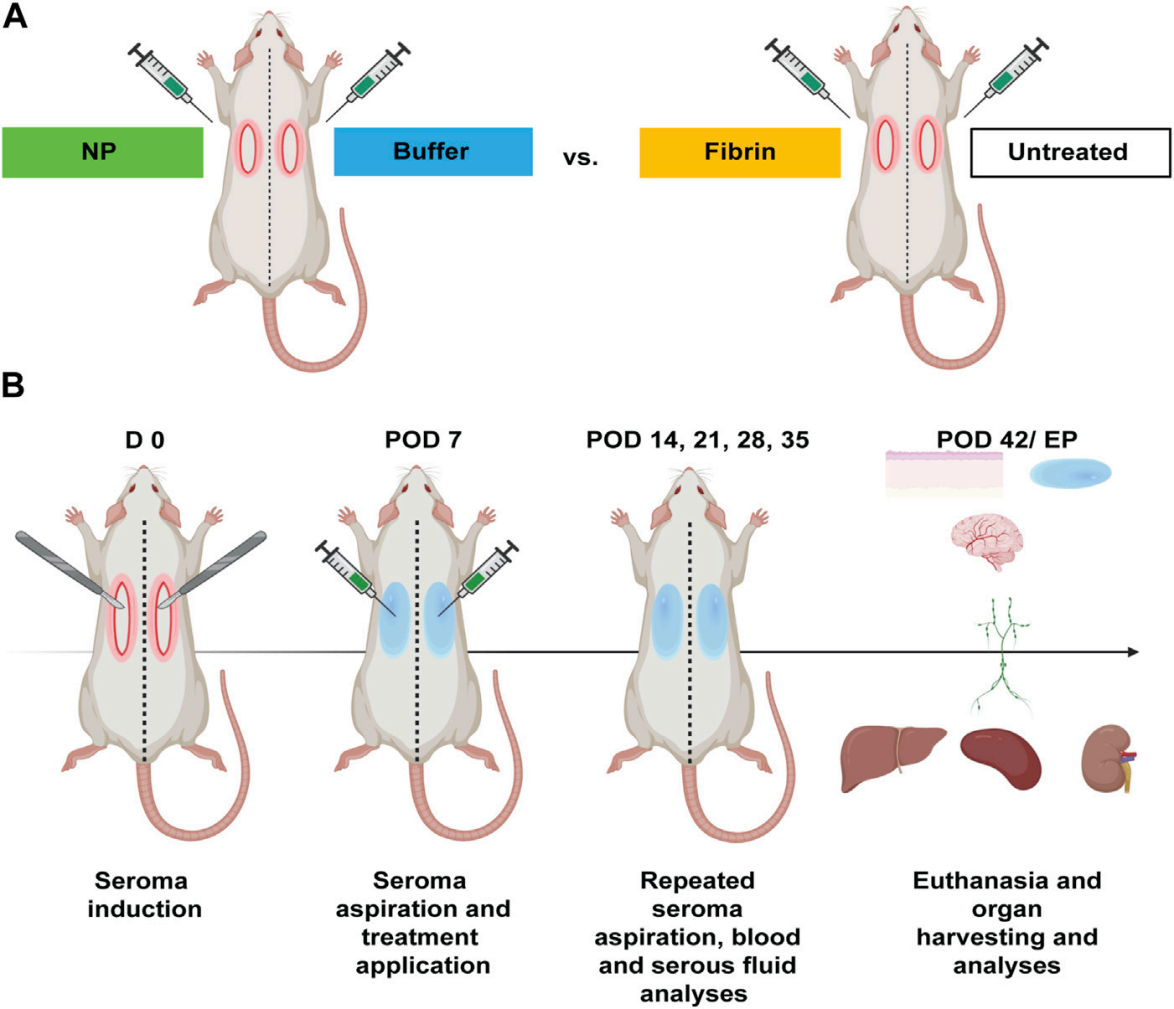
Experimental design for seroma induction, formation, aspiration, and treatments in Lewis rats (n = 20). **(A)** Seromas were surgically induced bilaterally in the axillary area of 20 Lewis rats (D 0). On POD 7 seromas were aspirated on the treatment-designated side, followed by application of zinc-doped strontium-substituted bioglass/ceria NPs in 10 rats and fibrin glue treatment in 10 rats. Control sides were treated with either buffer solution or left untreated. **(B)** Seroma fluid, blood and tissue samples were taken at defined timepoints. At euthanasia (POD 42/EP), NP systemic effects were assessed by blood analyses, and organ samples were analysed using biochemical, histopathological and immunohistochemical methods.

A total of 20 inbred male Lewis rats, weighing approximately 200–250 g were included in the experiment. After surgically inducing seromas on day 0, the seroma fluid was aspirated on postoperative day (POD) 7 and various treatment regimens were applied: in 10 rats, one side was injected into the seroma cavity with NPs dispersed in the given concentration in the buffer solution, while the other side was injected only with the buffer solution, a modified Ringer’s lactate buffer, containing citric acid and sodium citrate at 7.5 mM each; the other 10 rats underwent treatment with fibrin glue (Tisseel, Baxter AG, Opfikon, Switzerland) on one side, while the contralateral side was left untreated. The exact technique entailed aspirating the seroma fluid through a 22 Gauge sterile cannula after disinfecting the skin. Afterwards, while leaving the cannula in the cavity, a 1 mL sterile plastic syringe was attached to it and the assigned treatment regimen was injected through another 1 mL sterile plastic syringe. The skin enclosing the seroma cavity was left intact. The zinc-doped strontium-substituted bioglass/ceria nanoparticles-suspension contained 5 mg of NPs/1 mL of buffer solution. After trying out various concentrations, 1, 5, 10, and 15 mg of NPs, most efficacious first results were obtained using 5 mg of NPs. The same volume (1 mL) of fibrin glue, or buffer solution, was used for the other treatment groups (Figure 1A). Afterwards, at defined timepoints, seroma fluid was again aspirated if present, 0.5 mL of blood was drawn under anaesthesia, and various biochemical analyses (chapter 2.7.) were performed. At euthanasia (POD 42/endpoint (EP)), organs were harvested, and various tissue analyses (chapter 2.8., 2.9., and 2.10.) were performed (Figure 1B).

### 2.5 Elemental analysis of cerium (Ce) from the whole blood and organ samples

Systemic distribution of NPs in blood and to various organs, such as spleen, kidney, liver, brain, and lymph nodes (LNs), was quantified by elemental analysis of Ce using inductively coupled plasma mass spectrometry (ICP-MS) (Matter et al., 2020). To this end, whole blood (100 μL) or organ samples (50–300 mg) were transferred to a polytetrafluoroethylene container and mixed with 3 mL of 65% HNO_3_ p. a. (Merck) and 1 mL of 30% H_2_O_2_ p. a. (Merck). These samples were then digested using a microwave (TurboWAVE, MLS GmbH, Germany) at 250°C and 120 bar for 18 min. Cerium was then quantified using ICP-MS (Model 7900, Agilent, Santa Clara, CA) (Oertli et al., 1994; Kulber et al., 1997) by measuring isotope Ce140. Lu175 was used as an internal standard to correct for non-spectral interferences (Matter et al., 2017; Lese et al., 2021).

### 2.6 Systemic effects of NP treatment

Tests for the quantitative determination of blood urea nitrogen (BUN), creatinine, triglycerides, alanine aminotransferase (ALAT) and aspartate amino transferase (ASAT), were conducted on blood plasma collected at POD 0, 7, 14, 21, 28, 35 and 42 (Cobas c systems, Roche Diagnostics GmbH, Mannheim, Germany). These analytes represent biomarkers for organ failure (Matter et al., 2019). Analytes were read on the Roche Cobas 8,000 c system (Roche Diagnostics GmbH, Mannheim, Germany).

### 2.7 Cytokine measurements

Protein extraction from blood plasma, serous fluid, and skin tissue was performed as previously described (Valeta-Magara et al., 2015; Lese et al., 2018; Lese et al., 2021). Blood and serous fluid were collected at pre-defined timepoints (PODs 14, 21, 28, 35). The samples were spun down, twice for the blood, at 1,000 rcf for 10 min, followed by 1,500 rcf for 15 min, and once for the serous fluid at 1,500 rcf for 15 min (Valeta-Magara et al., 2015). Supernatants were collected and further subjected to quantitative assessments. 20–50 mg samples of skin were weighed on an analytical scale, cut into small pieces, and transferred to FastPrep^®^ Lysing Matrix tubes D, of 2 mL, containing 1.4 mm ceramic spheres (MP Biomedicals, Germany), and held on dry ice until lysate buffer was added. The lysate buffer contained 1x protease inhibitor cocktail (Halt Phosphatase Inhibitor Cocktail, Pierce, Rockford, IL, USA) and RIPA buffer (Pierce, Rockford, IL, USA), e.g., for 225 mg tissue, 2,250 µL RIPA buffer and 22.5 µL protease inhibitor were used. Tissue was then homogenized using the FastPrep-24 system (MP Biomedicals, Germany), followed by centrifugation at 13,000 rcf for 1 min at 4 °C. Each supernatant was then transferred to a new tube for further analyses.

Quantitative assessments of plasma, serous fluid and skin tissue analytes were performed using a customized commercial kit, Rat ProcartaPlex Mix & Match 9-Plex (Thermo Scientific, Bender MedSystems GmbH, Vienna, Austria) at defined timepoints for the following proteins: vascular endothelial growth factor A (VEGF-A), tumour necrosis factor alpha (TNF alpha), interleukin 1 beta (IL-1 beta), interleukin 2 (IL-2), interleukin 6 (IL-6), interleukin 10 (IL-10), monocyte chemoattractant protein-1 (MCP-1/CCL2), and interferon gamma (IFN gamma). TNF alpha, IL-1 beta, IL-2, MCP-1 and IFN-γ are known for their pro-inflammatory activity; IL- 10 is an anti-inflammatory cytokine. IL-6 is a cytokine that works both as pro-inflammatory and anti-inflammatory (Neligan, 2018). VEGF-A is a potent angiogenic cytokine, stimulating endothelial cell proliferation, playing a crucial role in angio- and vasculogenesis, regulated by all stages in the wound healing process, including inflammation (Esser et al., 1998; Howdieshell et al., 2001; Neligan, 2018; Lese et al., 2021). MCP-1, a monocyte chemoattractant, and INF gamma, a primary recruiter and activator of macrophages, are also known for their role in the inflammatory response (Neligan, 2018). The procedure was performed based on the original manufacturer’s instruction manual, and plates were read on a FLEXMAP 3D system (Luminex, Austin, CA, United States).

### 2.8 Histological analysis

At euthanasia (POD 42/EP), skin tissue was harvested and analysed. Some samples were rinsed in PBS, blotted dry and embedded in OCT matrix (Tissue-Tek, Sakura Finetek Europe BV, Leiden, Netherlands), and stored at −25 °C until sectioned (5 µm) using a cryostat. Other samples were rinsed in PBS, blotted dry and then fixed in formaldehyde until further histological processing.

Immunofluorescence (IF) on cryosections from OCT-embedded skin tissue samples, including the superficial capsule, was performed according to the methods of Zhang et al. (Zhang et al., 2018). Sections were fixed with acetone, followed by (double) primary staining with anti-CD31/PECAM-1 (dilution 1:100; AF3628-SP, bio-techne) and with each of the following antibodies: anti-CD68 (dilution 1:200; ab125212, Abcam), anti-MPO (dilution 1:250; A039829, Dako), anti-C3c (dilution 1:4,000; A0062, Dako), and anti-fibrinogen (dilution 1:100; A0080, Dako). Appropriate secondary antibodies [Alexa Fluor 568-labelled anti-rabbit IgG (A10042, 2,306,809, Invitrogen), and Alexa Fluor 488-labelled anti-goat IgG (A11055, 552,222, Molecular Probes)] were then used for imaging with a Zeiss LSM980 confocal microscope.

For histologic examinations, longitudinal sections of fixed skin were trimmed and routinely processed and stained with haematoxylin and eosin (H&E). Microscopic analysis was blindly performed by a board-certified veterinary pathologist (Dr. Simone de Brot). Slides were scanned (Nanozoomer S360 HAMAMATSU) and reviewed digitally (viewing software NDPview2 HAMAMATSU). Relevant histopathologic features were identified, and the following specific parameters were semi-quantitatively assessed: fibrosis, edema, vascularization, inflammation, presence of foreign material (NP and other).

Quantitative IHC analysis was performed digitally (Visiopharm 2022.11, Horsholm, Denmark). The capsule tissue was defined as a region of interest (ROI) and was fully quantified. CD68-positive cell counts were generated based on an automated deep learning classification. CD68 positive granulomas were manually labelled and measured (area in mm^2^). CD31-positive vessels were quantified and measured based on an automated deep learning classification. COL1 positive areas were quantified using a threshold classification based on the presence and intensity of staining (classified as dense vs less dense COL1 deposition).

### 2.9 Mass spectrometric skin tissue analyses

Frozen skin tissues were lysed in FastPrep^®^ Lysing Matrix tubes D, of 2 mL, containing 1.4 mm ceramic spheres, using a FastPrep-24™ 5G bead beating grinder and lysis system (MP Biomedicals, Germany) in approximately 500 µL lysis buffer: 8M urea/100 mM Tris-HCl pH 8, containing a proteases inhibitor cocktail (Complete EDTA free, Roche, Germany). After brief centrifugation, the supernatant was collected, and the beads washed with 300 µL lysis buffer and extra supernatant pooled with the previous one. Protein concentration was determined with Qubit Protein Assay (Invitrogen by Life technology, Zug, Switzerland). Then the extracted samples were reduced, alkylated, precipitated overnight, and stored at −20 °C until further use. The dry pellets were resuspended in 8M urea/50 mM Tris-HCl pH 8 to a protein concentration of 2 mg/mL. Samples of 10 µg protein were digested for shotgun data-dependent acquisition (DDA) and data-independent acquisition (DIA) processes, with LysC endopreoteinase for 2 h at 37 °C, followed by a digestion with trypsin at room temperature overnight, as previously described by Braga-Lagache et al. (Braga-Lagache et al., 2016).

These digests were analysed by nano-liquid chromatography tandem mass spectrometry on a system consisting of an Ultimate 3,000 (ThermoFischer Scientific, Reinach, Switzerland), coupled to a timsTOF Pro (Bruker Daltonics, Bremen, Germany), through a CaptiveSpray source (Bruker, Bremen, Germany) with an endplate offset of 500 V, a drying temperature of 200 °C, and with the capillary voltage fixed at 1.6 kV. A volume of 2 µL (200 ng) from the protein digest was loaded onto a pre-column (C18 PepMap 100, 5 μm, 100A, 300 µm i. d. X 5 mm length, ThermoFisher) at a flow rate of 10 μL/min with 0.05% TFA in water/acetonitrile 98:2. After loading, peptides were eluted in back flush mode onto a homemade C18 CSH Waters column (1.7 μm, 130Å, 75 μm × 20 cm) by applying a 90-min gradient of 5% acetonitrile to 40% in water/0.1% formic acid, at a flow rate of 250 nL/min. Each sample was analysed in DDA and DIA mode using the Parallel Acquisition Serial Fragmentation (PASEF) option. The mass range was set between 100 and 1700 m/z, with 10 PASEF scans between 0.7 and 1.4V s/cm^2^. The accumulation time was set to 2 m, and the ramp time was set to 100 m. Fragmentation was triggered at 20,000 arbitrary units (AU), and peptides (up to charge 5) were fragmented using collision induced dissociation with a spread between 20 and 59 eV.

DIA data was analysed by Spectronaut v16 (Biognosis) with factory settings and using a spectrum library generated by MSFragger (Yu et al., 2020) version 3.5 with the following parameters: database *Rattus norvegicus* from uniport (version 2022_03) (UniProt, 2019) enriched with common contaminants, MS1 and MS2 mass tolerances of ±20 ppm and ±0.05 Da, trypsin cleavage rule with a maximum of 3 missed cleavages, oxidation on methionine and protein N-terminal acetylation as dynamic modifications, carbamidomethylation of cysteines as fixed modification, respectively; the minimum of matched fragments was set to 6. PSMs validation was performed with the semi-parametric PeptideProphet model. The spectrum library was filtered from proteins identified by only one peptide before use with Spectronaut; the Spectronaut output was also filtered using this criterion.

Spectronaut protein intensity values were imputed in the following manner: if there was at most 1 detection in a replicate group, then the remaining missing values were imputed by a random draw from a Gaussian distribution of width 0.3 x sample standard deviation and shifted left from the sample mean mu by 2.5 x mu; all other missing values were replaced by the Maximum Likelihood Estimation method (MLE). Differential expression tests with moderated t-tests and significance criteria using 20 imputation cycles were performed as described by Uldry et al. (Uldry et al., 2022).

### 2.10 Scanning electron microscopy (SEM) imaging

Histological samples from serial formalin-fixed paraffin-embedded tissue sections of skin and superficial capsule tissue, stained for CD68, were analysed using Scanning Electron microscopy (SEM) combined with energy-dispersive X-ray spectroscopy (EDX) for element identification. To this end, the coverslips from the histological slides were removed using incubation in xylene for 3 days. After coverslip removal, the samples were air-dried and sputter-coated with 10 nm carbon and imaged using an Axia ChemiSEM (ThermoFisher Scientific). For image formation, a Backscattered Electron Detector (BSE Detector) was used. Cerium-containing particles were identified using a combination of this detector (mass contrast) and EDX spectroscopy (TrueSight LX Detector, ThermoFisher Scientific). Correlative light- and electron microscopy images were generated using the Software Maps (Maps 3.18, ThermoFisher Scientific).

### 2.11 Statistical analysis

GraphPad Prism, v9.5.1 (GraphPad Software, San Diego, California USA) was used for statistical analysis. All values are expressed as mean ± standard error. D’Agostino-Pearson and Shapiro-Wilk’s tests were used to assess data distribution. For normally distributed data, comparisons between different groups were performed using two-way ANOVA tests with Tukey *post hoc* analysis for multiple comparisons. For non-normally distributed data, the Wilcoxon signed-rank test was employed for comparisons among the same group, while the Mann-Whitney *U* test for single comparisons, and the Kruskal–Wallis test, with Dunn`s *post hoc* analysis for multiple comparisons, were used for the between-group analyses. *p* values < 0.05 were considered to indicate statistical significance.

## 3 Results and discussion

### 3.1 Early seroma volume reduction by NPs compared to other long-term conditions

**FIGURE 2:**
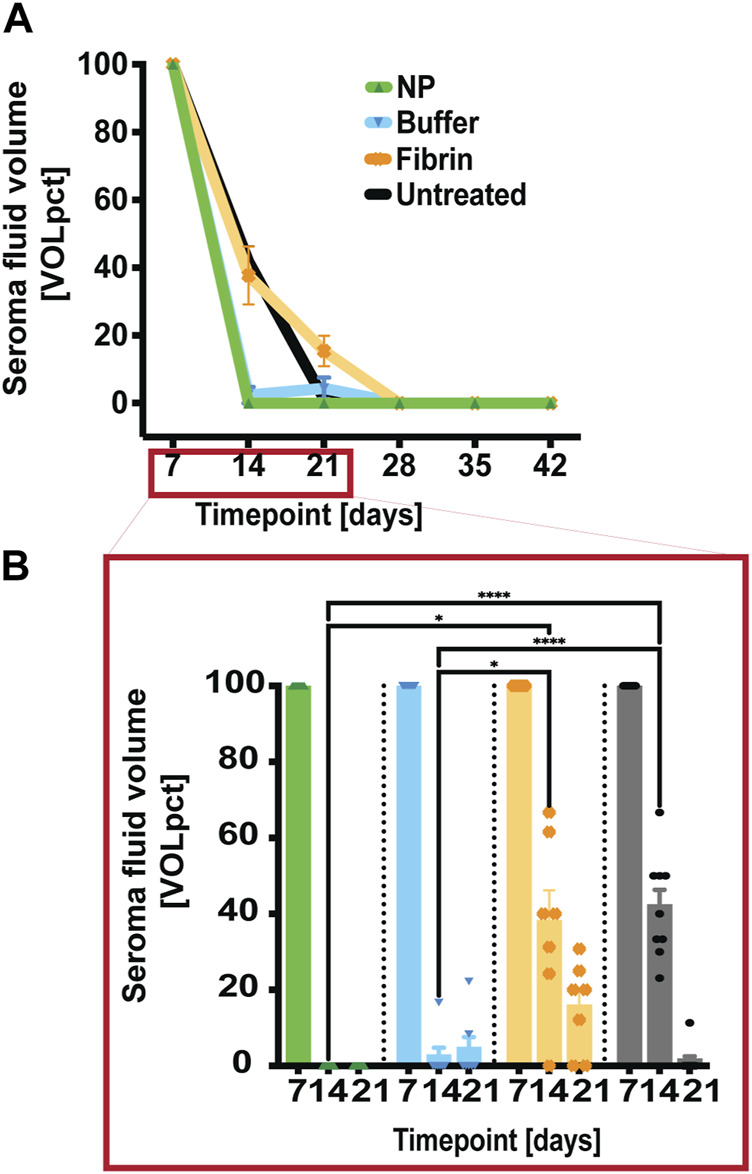
Early seroma reduction with NPs compared to long-term controls. **(A)** After seroma fluid aspiration on POD 7, the treatment sides were injected with either NPs or fibrin glue. At PODs 14, 21, 28, 35, and 42, seroma-fluid volumes were assessed after aspiration, and indicated as percentage of the recorded volume [VOLpct] **(B)** NP treatment resulted in significant early fluid reduction at POD 14 compared to the long-term fibrin glue group. Data = mean ± standard error of the mean. ****p < 0.0001, NPs and buffer solution vs untreated at POD 14. *p = 0.0176 and NPs vs fibrin glue, respectively *p = 0.0219, buffer solution vs fibrin glue at POD 14. Two-way ANOVA with Tukey *post hoc* for multiple comparisons.

To assess the effect of the NP-treatment on seroma volume, seromas were surgically induced in the rat model. At POD 7, bilateral postoperative seromas were observed in all rats. At this time point, seromas were aspirated (and the volume recorded) and injection of various treatment regimens were undertaken, depending on group allocation. Untreated-side seromas were only aspirated, and the volume recorded. At later timepoints (PODs 14, 21, 28, 35, and 42), fluid aspirates were also recorded (method description should be found in the experimental chapter). We observed a consistent reduction in fluid volumes over time in these long-term groups. Treatment with NPs showed the earliest and most complete volume reduction in this clinical model, with significant reductions (Figure 2) *versus* both the untreated (****p < 0.0001) and fibrin-treated (*p < 0.0176) sides at POD 14. However, similar results were also seen after treatment with buffer solution, with also significant reductions *versus* both the untreated (****p < 0.0001) and fibrin-treated (*p = 0.0219) sides at POD 14. It has previously been described that hypertonic saline solutions were used as sclerosants in preventing subcutaneous seroma formation (Dudai and Gilboa Ittah, 2019), which may explain the fact that buffer solution, containing Ringer`s lactate, citric acid and sodium citrate at 7.5 mM, could act as a hypertonic sclerosant. Nevertheless, NPs led not only to an early, but also complete seroma reduction at POD 14.

### 3.2 No long-term redistribution of NPs to the systemic circulation

To assess whether NPs were distributed systemically following application, blood analyses were performed using inductively coupled plasma spectroscopy (ICP-MS) to quantify elemental Ce (Matter et al., 2020).

**FIGURE 3:**
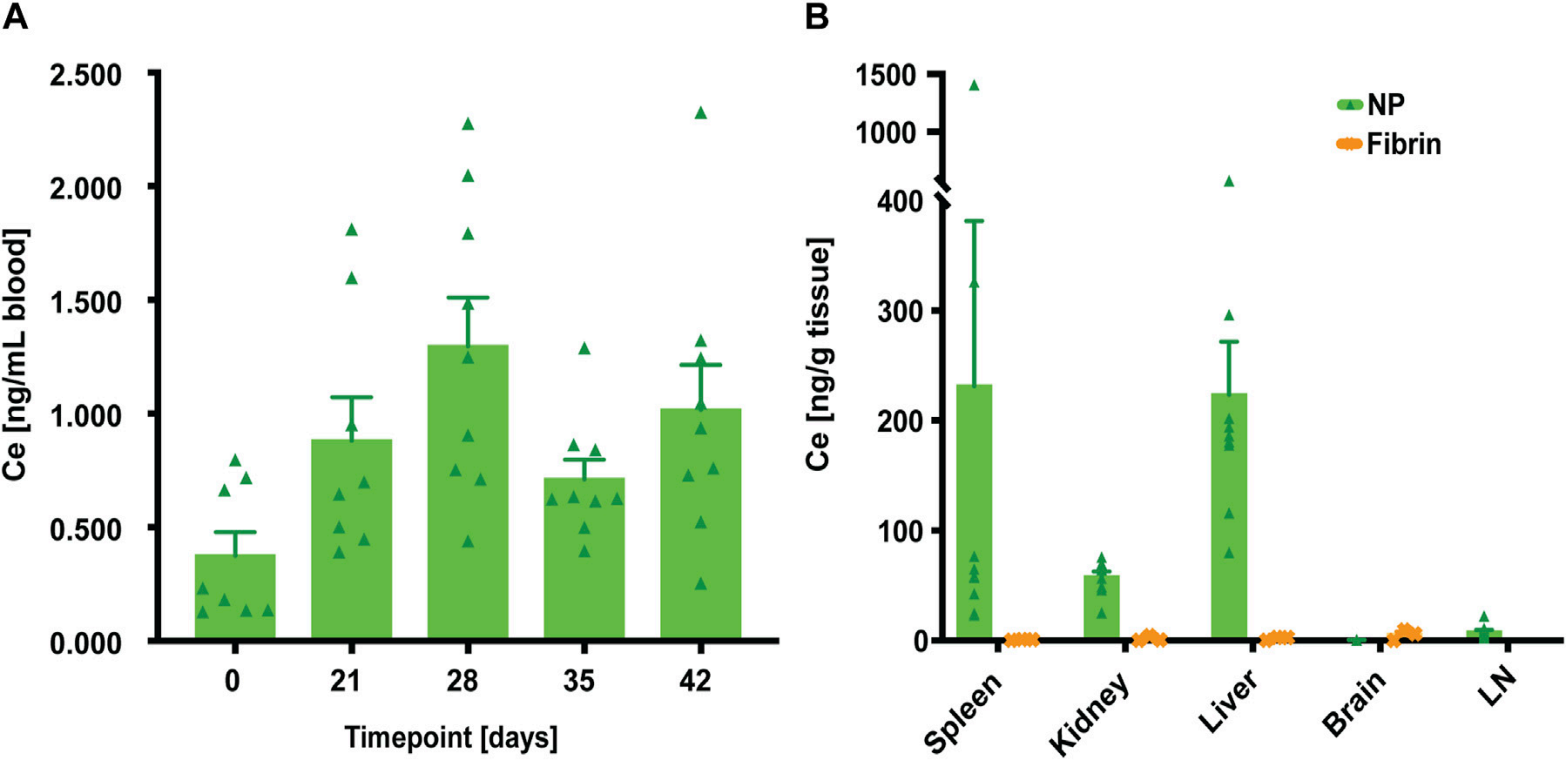
At euthanasia (POD 42), systemic distribution of NPs was quantified by elemental analysis of cerium (Ce) using inductively coupled plasma spectroscopy (ICP-MS). **(A)** Initial elemental analysis of Ce in the whole blood when compared to the biological baseline (POD 0) of untreated whole blood. Data = mean ± standard error of the mean. Kruskal–Wallis with Dunn`s *post hoc* for multiple comparisons indicated no significant differences between the different timepoints. **(B)** Analyses of Ce concentration in spleen, kidney, liver, brain, and LN tissue samples. For NP-treated rats (n = 9), the largest Ce concentrations were found in the liver. Data = mean ± standard error of the mean. Wilcoxon tests without significant differences between the groups.

For whole blood, diluted samples from defined timepoints (PODs 21, 28, 35 and 42) showed very low Ce levels when compared to the biological baseline (POD 0) of untreated whole blood, and rats which did not undergo surgery yet: median of 0.2 ng/mL (Figure 3). Highest values were observed on POD 28 (median of 1.24 ng/mL), and subsequently decreased, with values close to baseline at POD 35 (median of 0.62 ng/mL). To further assess if NPs also would distribute systemically to major organs, Ce concentrations from spleen, kidney, liver, brain, and LN, harvested at endpoint (POD 42), were determined using ICP-MS. Blood-rich organs such as spleen, kidney, and liver clearly showed increased Ce levels compared to the corresponding organs from fibrin treated animals. This might be explained by the already low levels of Ce quantified in whole blood. Furthermore, these Ce concentrations were considerably lower than the Ce background values of around 300 ng/g (i.e., untreated organs), reported by Park et al. (Park et al., 2009). When analysing both brain (median of 0.4 ng/g) and LNs (median of 6 ng/g), no increase in Ce levels after NP treatment was observed, suggesting that our NPs do not cross the blood-brain- and lymphatic barrier, respectively.

In agreement with our findings, assessing systemic distribution of NPs by ICP-MS, the elemental analysis of Ce showed virtually no clearance to the systemic circulation at endpoint (POD 42).

Plasma levels of organ-damage markers (BUN, creatinine, triglycerides, ASAT and ALAT) were also determined at defined timepoints to assess any long-term systemic effects of NP treatment (Supplementary Figure S1). We found no significant differences between groups, indicating that NPs did not cause any long-term systemic responses.

### 3.3 Inflammatory markers: NP treatment *versus* fibrin glue treatment

**FIGURE 4:**
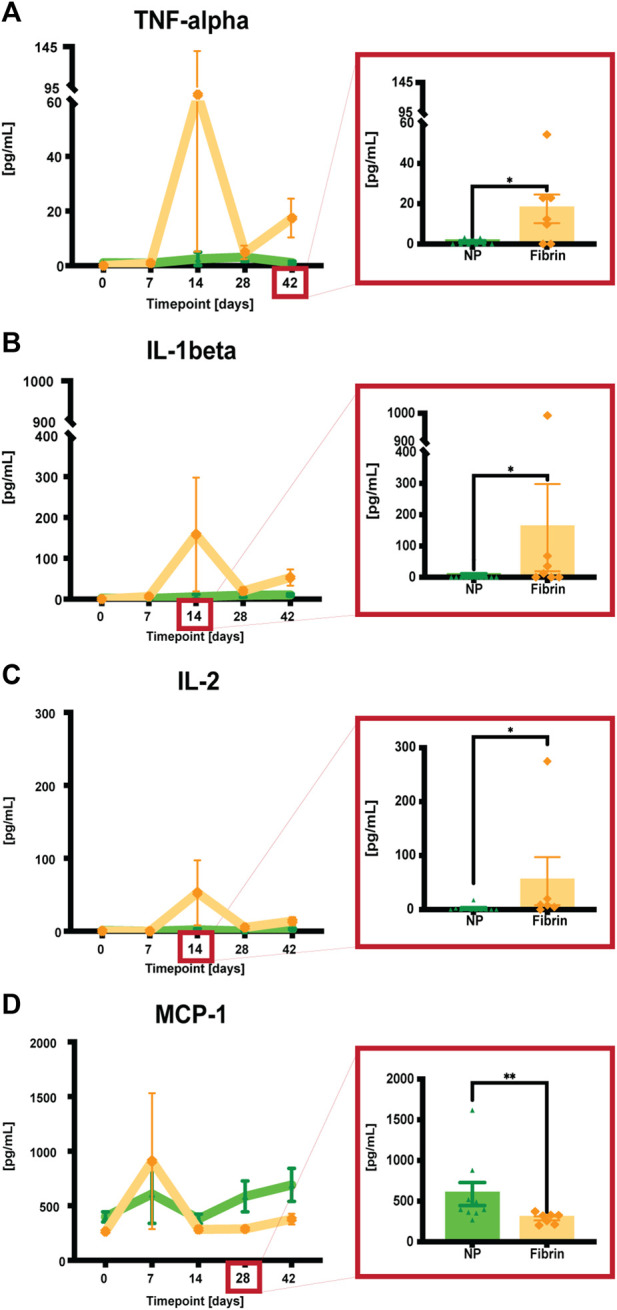
**(A-D)** Quantitative assessments of plasma analytes using a commercial kit (Luminex, BioPlex). When pg/mL values were not detectable (<3.17 for TNF-alpha, <9.45 for IL-1beta, <1.68 for IL-2, and <14.7 for MCP-1) they were assigned a bin value of 0. Data = mean ± standard error of the mean. Significant differences highlighted in red-framed close ups at the respective timepoints. *p = 0.028, TNF-alpha at POD 42, *p = 0.0294, IL1-beta at POD 14, *p = 0.014, IL-2 at POD 14, **p = 0.0048, MCP-1 at POD 28, NPs vs fibrin glue. Mann-Whitney tests for single comparisons. Two-way ANOVA with Bonferroni *post hoc* for multiple comparisons indicated no significant differences between the different treatments.

Following seroma-fluid aspiration/treatment at POD 7, we investigated the possibility of plasma NP-treatment responses by quantitatively assessing plasma analytes representing various inflammatory markers (e.g., VEGF-A, TNF alpha, IL-1 beta, IL-2, IL-6, IL-10, MCP-1, IFN gamma) at defined timepoints. We observed reduced levels of pro-inflammatory cytokines TNF-alpha, IL-1beta, and IL-2 after NP treatment compared to fibrin glue (*p = 0.028, TNF-alpha at POD 42, *p = 0.0294, IL1-beta at POD 14, *p = 0.014, IL-2 at POD 14). In contrast, MCP-1, a monocyte chemoattractant, was significantly increased (**p = 0.0048, MCP-1 at POD 28) in the long-term NP-treated group compared to the fibrin-glue group (Figure 4). A late-stage increase in MCP-1 concentrations may indicate the importance of late-stage macrophage recruitment for seroma formation.

We also examined the same analytes as inflammatory-response indicators in aspirated serous fluid. As the NP-treated group lacked this fluid after POD 14, only a comparison between the buffer solution, fibrin glue treated, and the untreated side was possible. The results indicated an increase in VEGF, IFN gamma, and MCP-1 levels after fibrin glue treatment, whereas TNF-alpha, IL-1beta, IL-2 and IL-6 were increased in the untreated group (Supplementary Figure S2).

When looking at skin tissue samples, together with the superficial capsule, as well as the deep capsule of the seroma at POD 42, VEGF, IL-1beta, and MCP-1 analyte levels were lower in the NP group compared to the other groups, with no significant difference between the groups (Supplementary Figure S3).

With the background knowledge that seroma formation is characterized by an inflammatory response due to surgical trauma (Watt-Boolsen et al., 1989; McCaul et al., 2000), we demonstrate that NP treatment exerts a long-term anti-inflammatory response in the blood plasma, significantly different compared to fibrin glue. These results validate therefore our group`s previous findings in the short-term seroma formation project (Lese et al., 2021). Also, they underline the adverse and pro-inflammatory effect after fibrin glue treatment, on the other hand. These findings may also help explain the long-term differences in seroma volume reduction between the different treatment groups, as fibrin glue took longest until seroma resolution. Therefore, NPs show promising and effective results for the treatment of seromas, when compared to the clinically widely used fibrin glue. This is being supported also by previous studies, which demonstrated that fibrin glue failed to show any benefit in minimizing seromas in a randomized clinical trial (Llewellyn-Bennett et al., 2012).

### 3.4 Long term, NP treatment led to cavity reductions and increased adhesions

**FIGURE 5:**
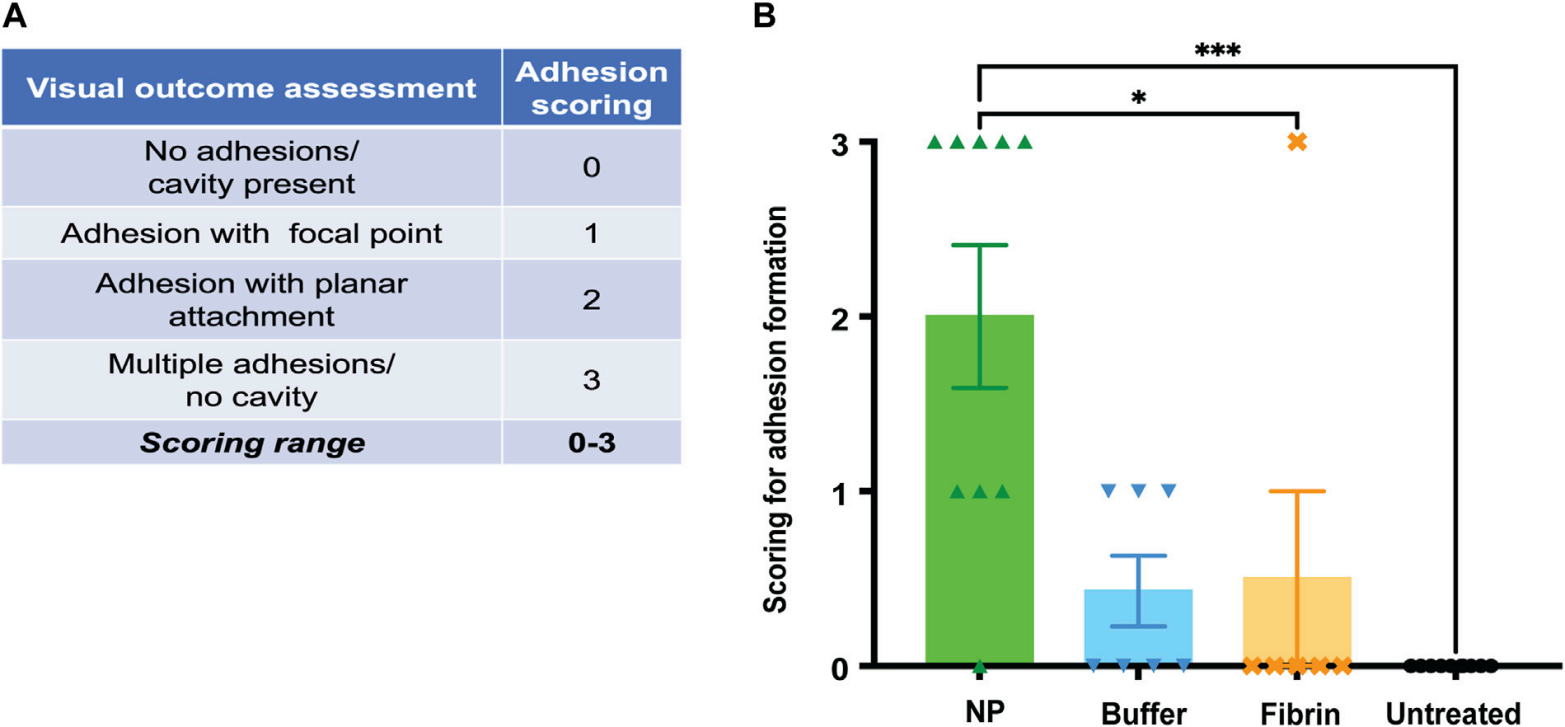
**(A)** At endpoint (POD 42), the surgical seroma sites were scored for macroscopic changes, including cavities and single/multiple adhesions with either focal or planar attachments. Scoring ranged from 0–3 points, with 0 representing no adhesions, but a cavity, and 3 representing multiple adhesions. **(B)** Complete resolution of cavities was observed in 5 out of 9 NP-treated rats. Data = mean ± standard error of the mean. Kruskal–Wallis with Dunn`s *post hoc* for multiple comparisons. ***p = 0.0004, NP vs untreated, and *p = 0.0358, NP vs fibrin glue.

As a next step, our goal was to quantify the extent of adhesion formation. Rats were euthanized at endpoint (POD 42), and the surgical sites were excised for further macroscopic observations, based on previous findings regarding formation of adhesions (Rodgers and diZerega, 1993; Chegini et al., 2002; Ergul and Korukluoglu, 2008; Maciver et al., 2011; Coccolini et al., 2013; Fang et al., 2018; Vediappan et al., 2020) after tissue trauma. We performed a macroscopic assessment of adhesion formation and envisioned a scoring system raging from 0 to 3 (Figure 5). This analysis showed that NP-treated sides featured significant macroscopic changes, including increased adhesive properties and complete cavity closure, compared to non-NP treated sides (***p = 0.0004, NPs vs untreated sides, and *p = 0.0358, NPs vs fibrin sides) (Figure 5).

### 3.5 Increase in macrophage recruitment after NP treatment as demonstrated by immunofluorescence

Based on principles of wound healing and peritoneal adhesion formation (Gurtner et al., 2008; Maciver et al., 2011; Neligan, 2018), different stages of seroma formation and adhesions are likely an early inflammatory process involving neutrophil recruitment that soon shifts to macrophages, followed by activation of the coagulation cascade. With NP treatment, the environment would favour both an abundant fibrin/collagen matrix and angiogenesis representing a fibrin-fibrinolysis-disbalance (Maciver et al., 2011). Treatment with NPs would finally lead to adhesion formation, primarily accompanied by depositions of extracellular substance, presumably collagen. Late-stage macrophage infiltration may also be associated with adhesion formation (Maciver et al., 2011).

**FIGURE 6:**
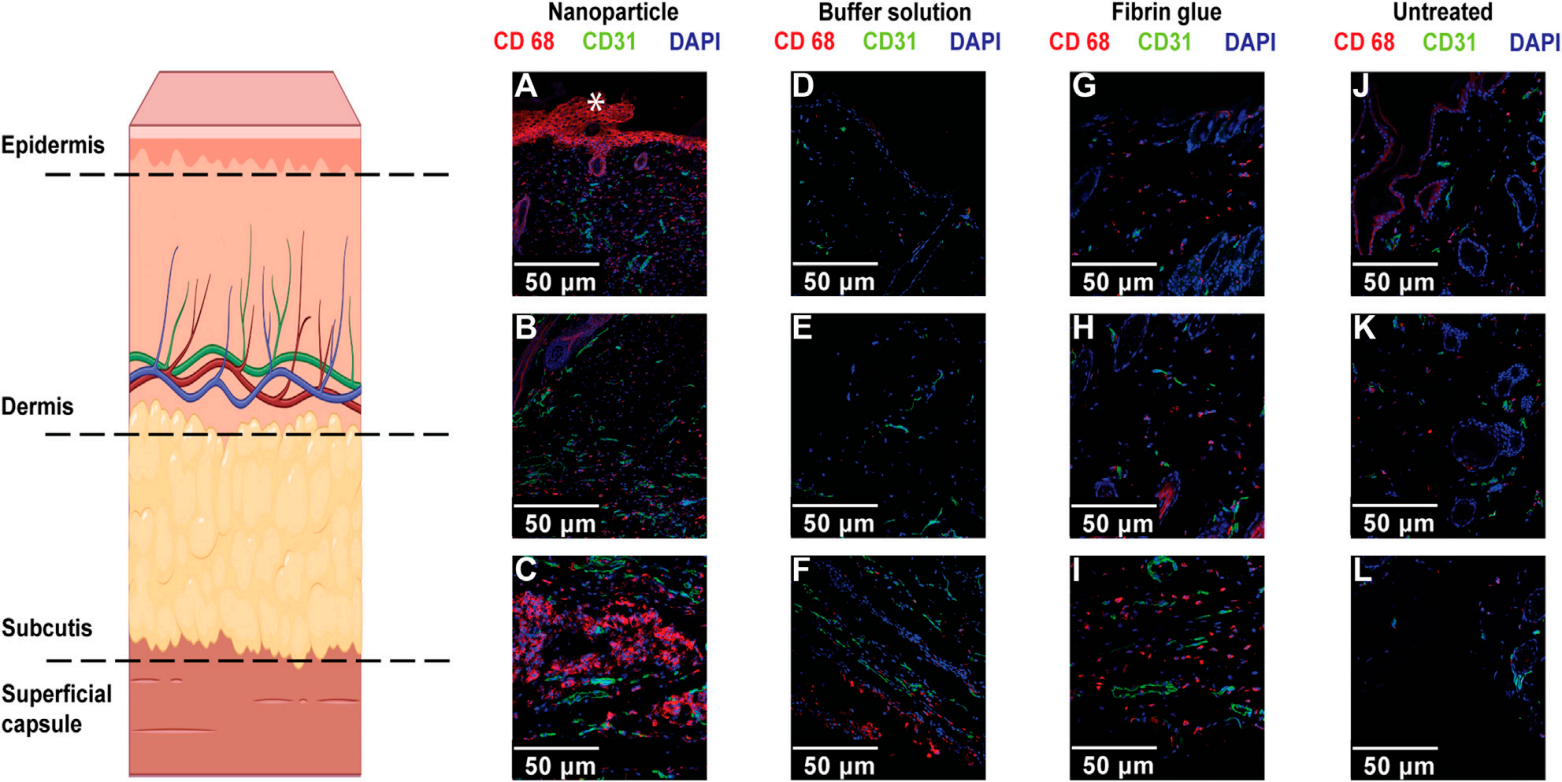
Confocal microscope imaging of IF staining for macrophages (CD68) and endothelial cells (CD31), with DAPI nuclear staining in skin/capsule tissue of NP treated **(A–C)**, buffer treated **(D–F)**, Fibrin treated **(G–I)** and untreated groups **(J–L)**. Single position pictures of epidermal, intradermal, and basal structures (scale bars, 50 μm). Increased staining intensity for CD68^+^ macrophages (co-localized with nuclei) was mostly observed within skin basal regions after NP treatment and increased staining for endothelial cell was mostly observed in NP-treated and untreated sides compared to the control group. Non-specific staining (*) in the epidermis and hair follicles was also observed.

We therefore decided to use immunocytochemistry to label crucial cell markers and structural proteins during these different stages of seroma/adhesion formation. Figure 6 shows IF stainings of cross-sections of skin tissue after NP, buffer solution, fibrin glue treatment and of the untreated side. Our analyses consisted of looking at the following markers: CD68 (for macrophages), CD31 (for endothelial cells of vessels), and blue DAPI representing cell nuclei.

We concluded that there was an increase in labelled macrophages, identified as CD68, after NP treatment, basally alongside the superficial capsule compared to the control groups (Figure 6). These findings are consistent with an ideal seroma-forming environment, but also favour adhesion formation and thereby favour seroma cavity closure. It is interesting to note that increased macrophage staining via CD68 was also present in the skin of untreated long-term controls, but in all cases, this was considerably less prominent than that seen with NP treatment, highlighting late-stage macrophages infiltration and its potential role in NP uptake and finally, seroma resolution.

**FIGURE 7:**
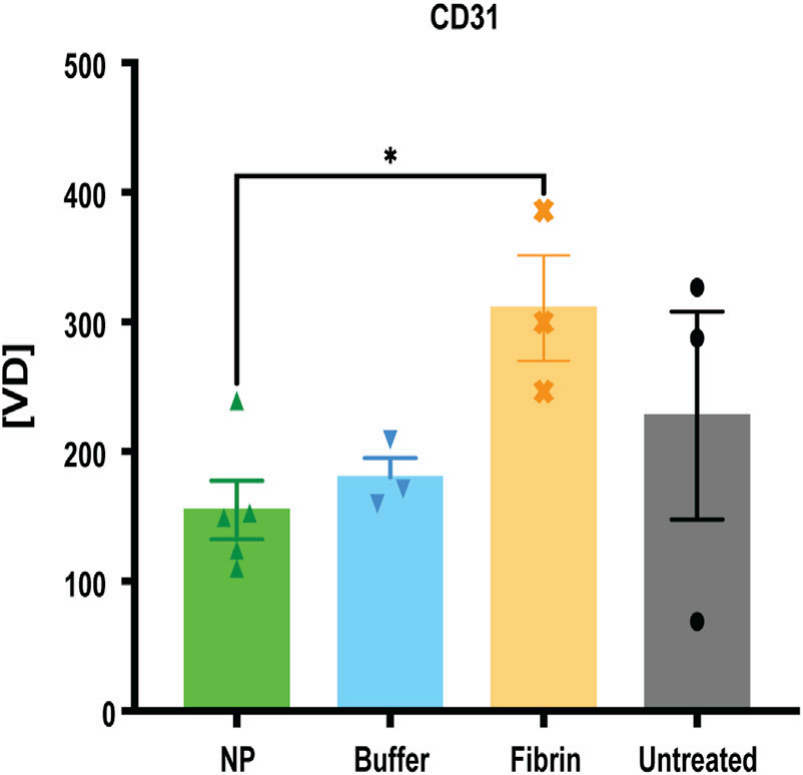
Decreased vascularization after NP treatment. IF staining for CD31, determining CD31 positive vessel count, indicated as vessel density [VD]. Data = mean ± standard error of the mean. *p = 0.0357, NPs vs fibrin glue, Mann-Whitney tests for single comparisons. Kruskal–Wallis tests with Dunn`s *post hoc* for multiple comparisons with no significant differences between the different treatments.

Additionally, we observed a significant decrease in labelled endothelial cells, represented as CD31, in the superficial capsule tissue, after treatment with NPs, compared to fibrin glue (*p = 0.0357) (Figure 7). It is well known that endothelial cells are essential for wound healing and maturation, regulated by different stages of inflammation and skin wound angiogenesis (Gurtner et al., 2008; Neligan, 2018). In regard to our previous findings that NPs exert a long-term anti-inflammatory response, our current results with decreased vascularization after NP treatment, underline the detrimental pro-inflammatory effect of fibrin glue (Lese et al., 2021).

### 3.6 Increase in macrophage recruitment after NP treatment as demonstrated by immunohistochemistry

Relevant features of seroma formation, especially macrophage infiltration, vascularization, and deposition of COL1, were evaluated using mass spectrometric, proteomic and immunohistochemical analyses of the skin and superficial capsule tissue run after euthanasia (EP/POD 42) (Supplementary Figure S4). Tissues, e.g., skin/superficial capsule, were harvested, processed, and analysed further via mass spectrometry at endpoint (EP). From the 2,274 identified proteins we selected 26 structural proteins of interest in the process of seroma and adhesion formation (Gurtner et al., 2008; Maciver et al., 2011; Neligan, 2018). Even if there were no significant differences between the groups, different clusters of abundance were observed for the selected proteins, especially an enrichment in macrophages and COL1, with potential importance in late-stage differentiation, respectively maturation (Schroder et al., 2010; Kubala et al., 2018; Kubala and DeClerck, 2019).

Additionally, when skin and superficial capsule tissues were analysed using IHC, we specifically examined the levels of macrophage infiltration and granuloma formation (CD68), vascularization (CD31) and level of collagen depositions (COL1).

**FIGURE 8:**
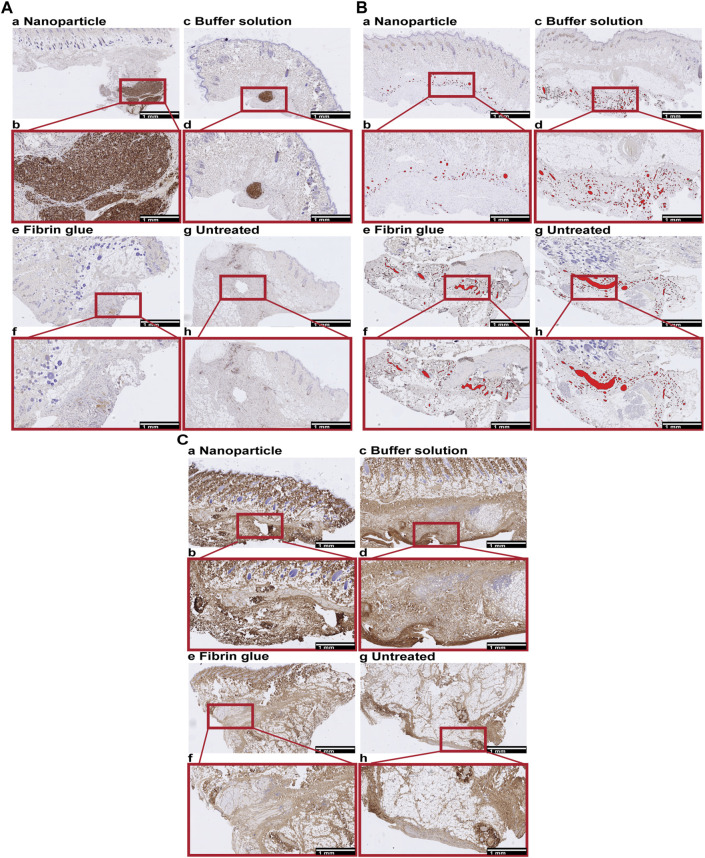
Immunohistochemical (IHC) staining for macrophages (CD68), vessels (CD31) and collagen type 1 (COL1) in skin, containing the superficial seroma capsule of NP treated **(a,b)**, buffer treated **(c,d)**, fibrin glue treated **(e,f)**, and untreated groups **(g,h)**. Overview and enlarged areas at the capsular level of skin tissue sections. Scale bars, 1 mm. **(A)** CD68. Nodular dense aggregates of CD68 positive cells correspond to granulomas which are most extensive after NP, small and focal after buffer solution treatment and absent in the fibrin glue treated and untreated side. **(B)** CD31. CD31 positive vessels are labelled in red. The level of vascularization is lower in NP compared to other treatment groups. **(C)** COL1. Collagen deposition tended to be multifocal and to be denser after NP treatment compared to other treatment groups.

**FIGURE 9:**
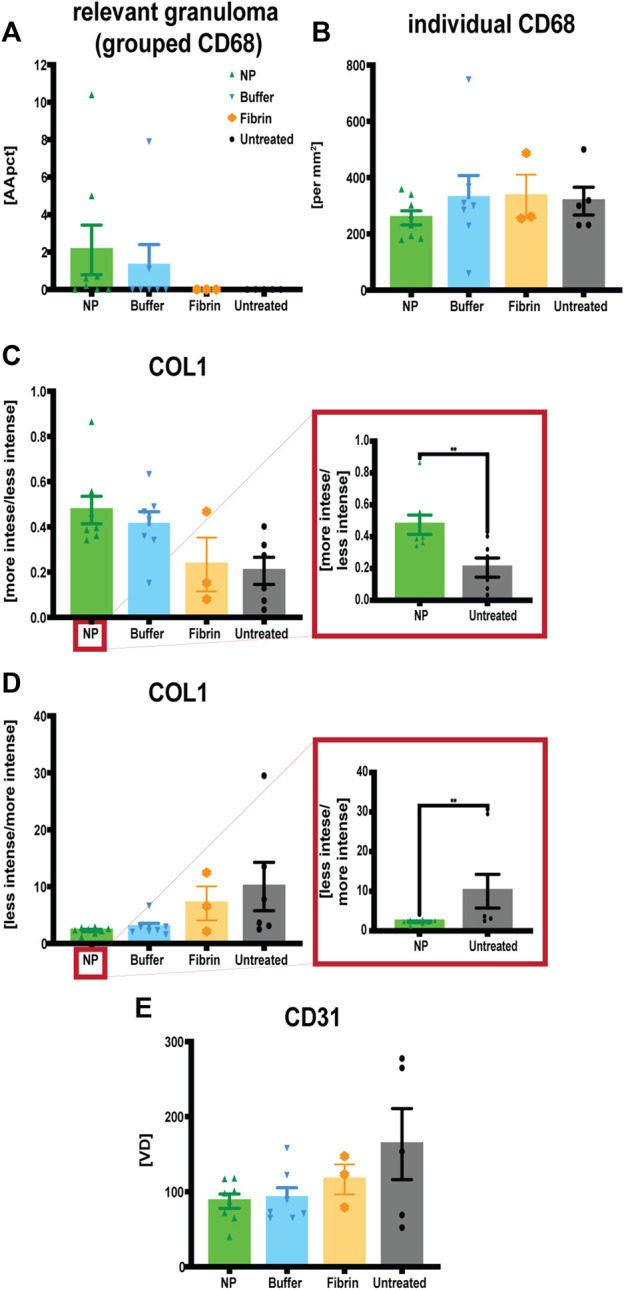
Quantitative assessments of immunohistochemistry staining for macrophages (CD68), endothelial cells (CD31) and collagen type 1 (COL1) in capsule tissue, comparing four treatment conditions: NP vs buffer vs fibrin glue vs untreated. **(A)** Increased granuloma formation (grouped macrophages) after NP treatment. Area of CD68 positive stained granuloma was measured and indicated as percentage of the assessed capsule tissue [AApct]. Data = mean ± standard error of the mean. **(B)** Decreased individual macrophages after NP treatment indicated as cell counts [per mm^2^]. Data = mean ± standard error of the mean. **(C)** Increases in more intense collagen type 1 deposition after NP treatment. IHC staining for COL1 measuring the ratio between more vs less intensely stained (i.e., densely arranged) collagen [more intensity/less intensity]. Data = mean ± standard error of the mean. Mann-Whitney tests. **p = 0.0221, NP vs untreated, *p = 0.028, buffer vs untreated, Mann-Whitney tests for single comparisons. **(D)** Reduction in less intense COL1 deposition after NP treatment. IHC staining for COL1 measuring the ratio between less vs more intensely stained (i.e., densely arranged) collagen [less intensity/more intensity]. Data = mean ± standard error of the mean. **p = 0.0221, NP vs untreated, *p = 0.028, buffer vs untreated, Mann-Whitney tests for single comparisons. Kruskal–Wallis tests with Dunn’s *post hoc* for multiple comparisons indicated no significant differences between the different treatments. **(E)** Decreased vascularization after NP treatment. IHC staining for CD31, determining CD31 positive vessel count, indicated as vessel density [VD]. Data = mean ± standard error of the mean.

IHC analyses of CD68, CD31 and COL1 showed increased granuloma formation (CD68^+^) and denser COL1 deposition within seroma capsule tissue after NP treatment compared to the control group, but also decreased vascularization after NP treatment (Figure 8; Figure 9). The assessed area corresponded to the seroma capsule and adjacent dermal tissue, excluding muscle, fat, and glands.

Granuloma formations (i.e., area of macrophage groupings [AApct]) were histologically mainly observed for three main reasons, associated with: broken hair shafts, phagocytosis of NPs, or without any visible foreign material. Broken hair shafts were considered unrelated to treatment with NPs. This situation has therefore been indicated separately. Indeed, relevant granuloma formation has been restricted to NPs. There is clear evidence that macrophages take up NPs and form granulomas in the seroma capsule (Figure 8A).

On the other hand, when comparing counts of individual macrophages [per mm^2^], lowest values were however observed after treatment with NPs. This underlines the long-term anti-inflammatory tissue effect of NPs.

NPs exert an anti-inflammatory response both in the blood plasma, as previously shown in chapter 3.3, and capsule tissue. Similar results were also obtained by Legon`kova et al. and Gong et al. in wound repair, where they showed that Ce-based nanomaterials stimulate the wound healing and epithelial regeneration process in the presence of lymphocyte–macrophage infiltration (Legonkova et al., 2017; Gong et al., 2022).

Further analysis of COL1 deposition based on density used a comparison of ratios between histologically looser vs denser collagen, and *vice versa*. We observed an increase in dense COL1, and at the same time, a reduction in loose COL1 deposition after NP treatment (Figure 8C; Figure 9). Those differences after application of NPs resulted in significant differences compared to the untreated side (**p = 0.0221). Even if buffer solution treatment showed similar significant differences when compared to the untreated side (*p = 0.028) (Dudai and Gilboa Ittah, 2019), other than after NP treatment, when looking at the overall macroscopic picture, it did not lead to complete adhesion formation, cavity closure, and seroma remission (Figure 7).

The presence of more densely arranged collagen at the capsular level is highly suggestive for a more compact and connective collagen and suggests an environment abundant in matured collagen after NP treatment and indicating their adhesive properties (Rodgers and diZerega, 1993; Chegini et al., 2002; Ergul and Korukluoglu, 2008; Maciver et al., 2011; Coccolini et al., 2013; Fang et al., 2018; Vediappan et al., 2020). On the other hand, the less dense COL1 depositions are indicative of a looser type of fibrous connective tissue, potentially indicating a fibrin-fibrinolysis-disbalance, in combination with late-stage macrophage recruitment (Maciver et al., 2011).

### 3.7 Co-localization of macrophages with NPs using correlative microscopy

**FIGURE 10:**
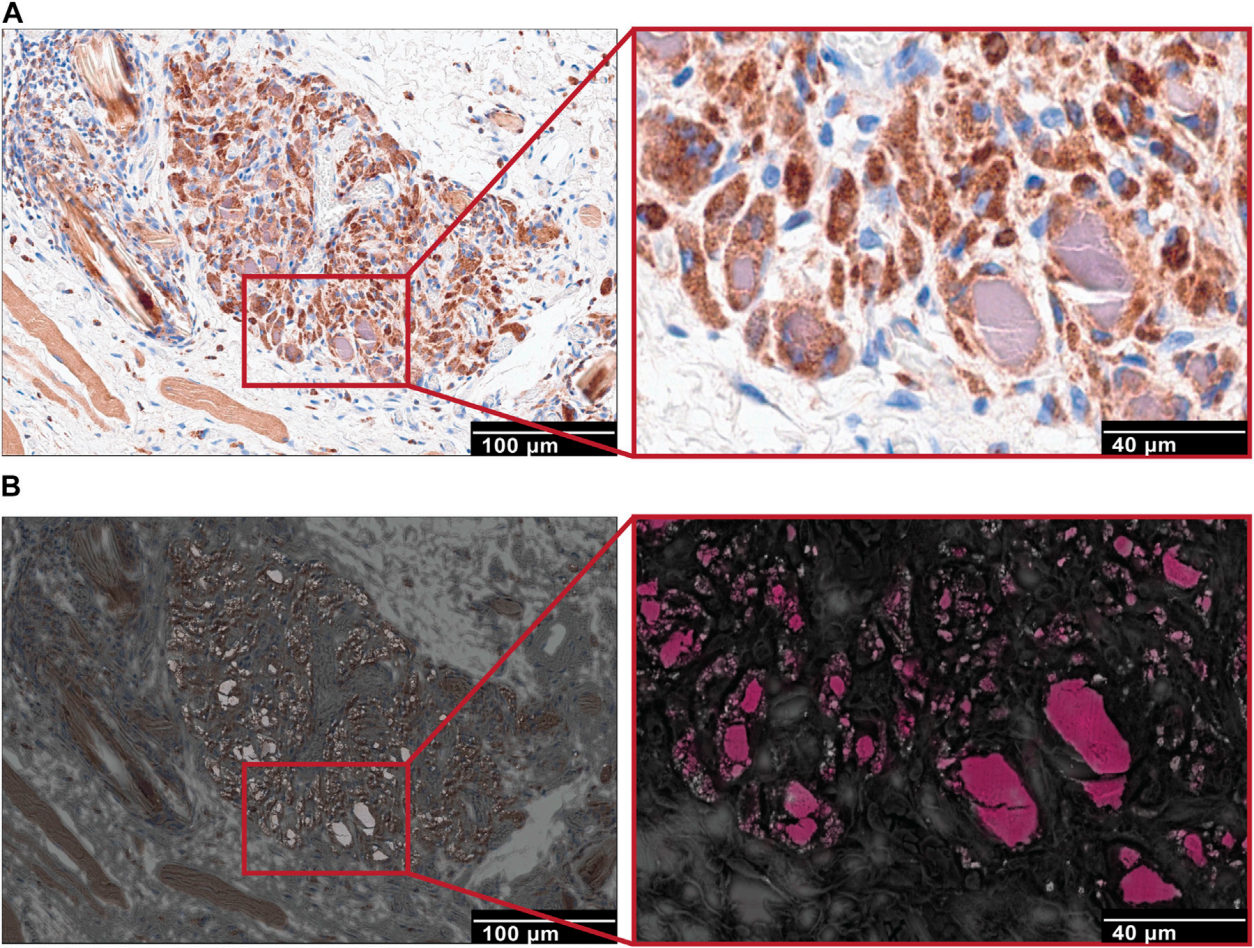
Correlation between IHC and SEM imaging techniques. **(A)** Immunohistochemistry staining for macrophages (CD68) in capsule tissue of NP treated groups. Focal granuloma with CD68 positive stained macrophages that contain intracellular purple-grey substance (NP accumulation). Scale bar, 100 μm. Higher magnification, scale bar, 40 μm. **(B)** SEM images of the corresponding to immunohistochemical images, collected using a backscattering detector (A.). Electron dense region sections (scale bars, 100 μm) and maps indicating cerium represented in pink color (Ce).

As a next step, when using correlative techniques between immunohistochemical and Scanning electron microscopy (SEM) analyses, we could observe an evident co-localization of macrophage staining with NPs, indicating the role of macrophages in the uptake and hence retention of NPs at the site of application (Figure 10).

## 4 Limitations

Here, we highlight the role of late-stage macrophage infiltration and COL1 depositions after NP treatment that may lead to adhesion formation and cavity closing. However, the exact pathways regulating seroma formation and resolution remain complex. Therefore, future molecular studies of the pathophysiology of seroma formation and the mechanism of action of NPs are still important.

The buffer solution (lactated Ringer’s with citric acid and sodium citrate) also showed sclerosant properties (Dudai and Gilboa Ittah, 2019) similar to NPs, but at a later timepoint and macroscopically not with cavity obliteration. This buffer formulation should be re-examined, and potentially revised. The combined application though, of NPs and buffer solution together, may have a more potent sclerosant effect for seroma cavity closure.

The development of a large animal model for seroma formation is crucial for preclinical testing. Using it for comparing NPs to a gold standard such as fibrin glue would better validate the clinical value and effectiveness of bioactive inorganic NPs.

## 5 Conclusion

These results demonstrate the ability of inorganic nanoparticle-based formulations to reduce seroma formation in a previously established rat model. Although several studies have explored the use of non-surgical sclerosant treatments for seroma, such as methylprednisolone (Qvamme et al., 2015), *Mytilus edulis* protein (Chung et al., 2006), lysine-derived urethane (Gilbert et al., 2008), and tranexamic acid (Oertli et al., 1994), standardized care protocols and management recommendations in clinical settings remain lacking. Moreover, while prior research demonstrated only partial seroma reduction with other sclerosants, our current study showcased complete seroma resolution following NP treatment. Treatment with NPs also showed a significant anti-inflammatory response compared to fibrin glue, without any detectable systemic adverse effects. Macroscopically, at the site of NP application, we observed increased adhesion formation which obliterated the seroma cavity long term. These findings stand in contrast to the commonly used fibrin glue, which demonstrates pro-inflammatory properties and appears less promising in minimizing postoperative seroma formation. While the pathogenesis of seroma remains poorly understood, our research has elucidated pivotal mechanisms governing the pathophysiological processes underlying seroma formation. Microscopically, we observed late-stage macrophage infiltration, and collagen type 1 depositions alongside the superficial capsule and underlying the capsule, emphasising the crucial role of macrophages and COL1 in adhesion formation and reducing seroma recurrence risk. Macrophages did clearly co-localize with NPs, suggesting the uptake and retention of NPs at the treatment site. This long-term study supports the use of bioactive inorganic nanoparticles for the safe and efficacious therapeutic management of seroma.

## Data Availability

The original contributions presented in the study are included in the article/Supplementary Material, further inquiries can be directed to the corresponding author.
