# Supplementary material for: Bioglass/ceria nanoparticle hybrids for the treatment of seroma: a comparative long-term study in rats
Source: Front Bioeng Biotechnol. 2024 Mar 12;12:1363126. doi: 10.3389/fbioe.2024.1363126 (PMC10963406; doi:10.3389/fbioe.2024.1363126)
Supplement: Supplementary file 1 [file Table1.DOCX]

***Supplementary Material***

**Bioglass/ceria nanoparticle hybrids for the treatment of seroma: a comparative long-term study in rats**

**Michael-Alexander Pais*, Athanasios Papanikolaou, Isabel Arenas Hoyos, Robert Nißler, Simone de Brot, Alexander Gogos, Robert Rieben, Mihai A. Constantinescu, Martin T. Matter, Inge K. Herrmann, Ioana Lese**

*** Correspondence:** Michael-Alexander Pais: [michaelalexander.pais@gmail.com](mailto:michaelalexander.pais@gmail.com)

Suppl. Fig. 1 A. Plasma levels of organ-damage markers: NP treatment versus fibrin-glue treatment. Blood plasma assessments of BUN, creatinine, triglycerides, ASAT, and ALAT levels were made at defined timepoints. Data = mean ± SEM. Kruskal-Wallis tests with Dunn`s post-hoc for multiple comparisons indicated no significant differences between the groups.

Suppl. Fig. 2 Serous fluid inflammatory markers: fibrin glue treatment versus untreated. The lack of seroma fluid in the NP-treated group after POD 14 meant that only fibrin glue vs. buffer solution vs. untreated comparisons were possible. At PODs 14, 21, 28, 35, and 42, quantitative assessments of seroma fluid cyto- and chemokines (A-H) were performed using a commercial kit (Luminex, BioPlex). When pg/ml values were not detectable (<5.45 for VEGF, <3.17 for TNF-alpha, <9.45 for IL-1beta, <1.68 for IL-2, <2.12 for IL-6, <6.25 or for IL-10, <3.27 for INF-gamma, and <14.7 for MCP-1) they were given a bin value of 0. Data = mean ± SEM. Kruskal-Wallis tests with Dunn`s post-hoc for multiple comparisons indicated no significant differences between groups.

Suppl. Fig. 3 NP treatment indicated less of an inflammatory response in deep-capsule tissue compared to the untreated side. Biochemical analyses of VEGF, IL-1beta, INF-gamma, and MCP-1 in skin/superficial capsule (A-C) and deep capsule tissue samples (D-F) harvested at endpoint (POD 42). When pg/ml values were not detected (<5.45 for VEGF, <9.45 for IL-1beta, and <14.7 for MCP-1) they were given a bin value of 0. Data = mean ± SEM. Kruskal-Wallis tests with Dunn`s post-hoc for multiple comparisons. The Kruskal-Wallis tests used for VEGF, IL-1beta, and MCP-1 levels from both skin/superficial capsule and deep capsule showed no significant differences between groups.

**Suppl. Fig. 4** NP treatment indicated an increased abundance of collagen type I in skin and superficial capsule tissue compared to the control groups. Mass spectometric analyses of protein abundance in skin/superficial capsule tissue samples (n = 5 per each treatment group) harvested at endpoint (POD 42), comparing four treatment conditions: NP vs. buffer vs. fibrin glue vs. untreated. Color-coded heat map representation of the abundance of four selected proteins of interest for seroma formation. Highest values are yellow, and intensities were log2 transformed. Data = mean. Kruskal-Wallis with Dunn`s post-hoc for multiple comparisons indicated no significant differences between the different treatments.
