# Supplementary figures and images for: Bioglass/ceria nanoparticle hybrids for the treatment of seroma: a comparative long-term study in rats
Source: Front Bioeng Biotechnol. 2024 Mar 12;12:1363126. doi: 10.3389/fbioe.2024.1363126 (PMC10963406; doi:10.3389/fbioe.2024.1363126)

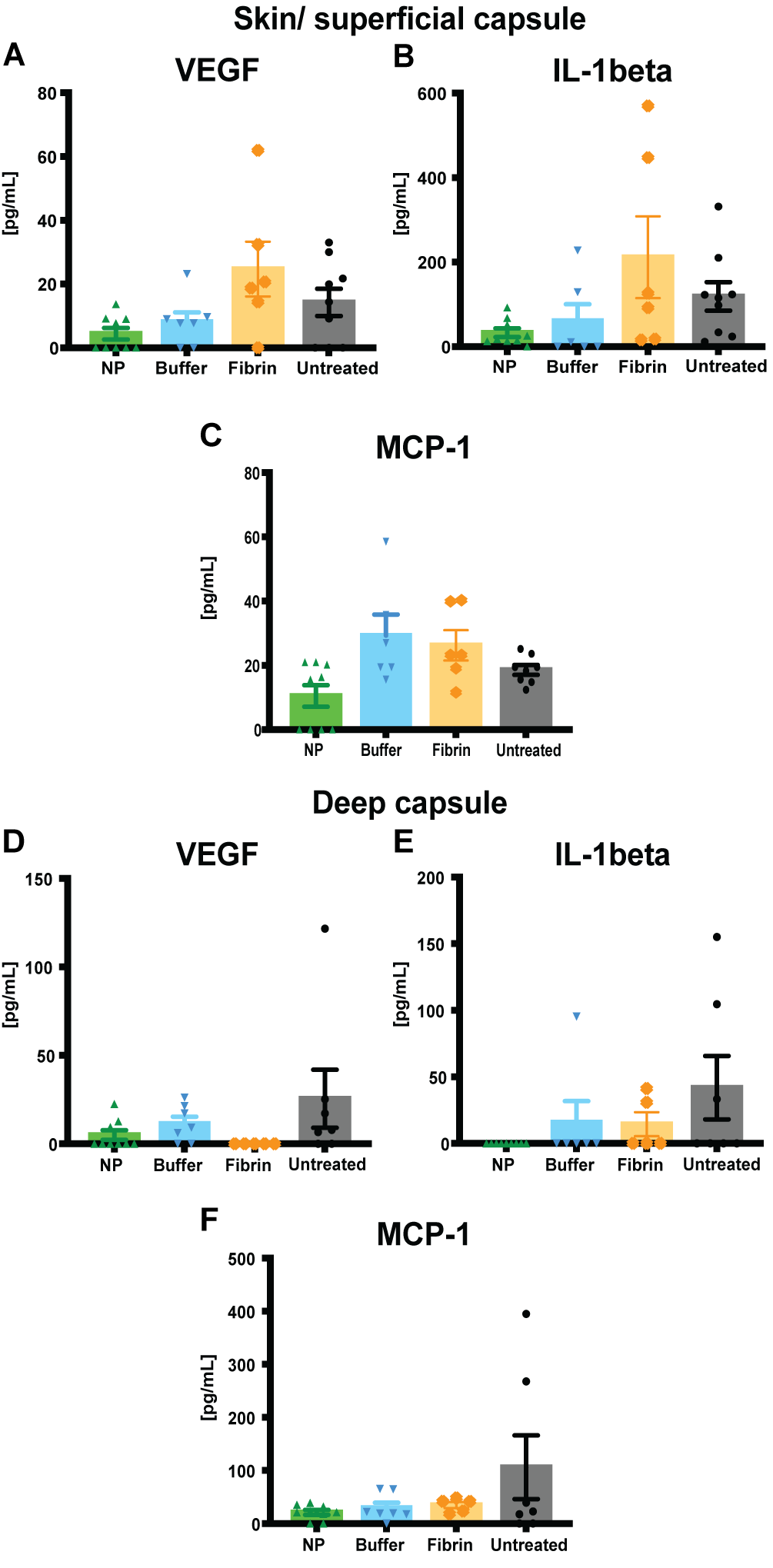

Supplement: Supplementary file 2 [file Image3.tif]

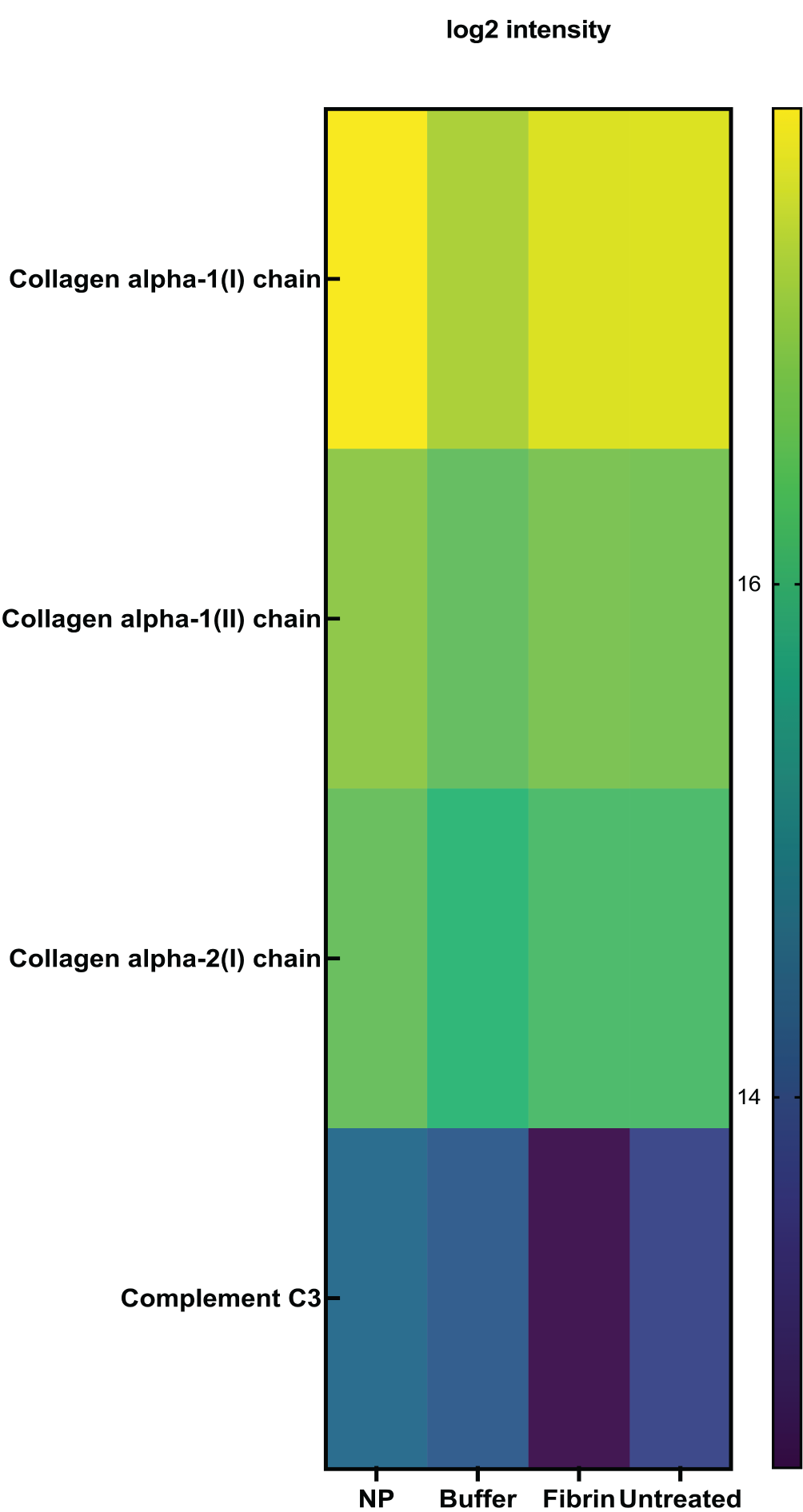

Supplement: Supplementary file 3 [file Image4.tif]

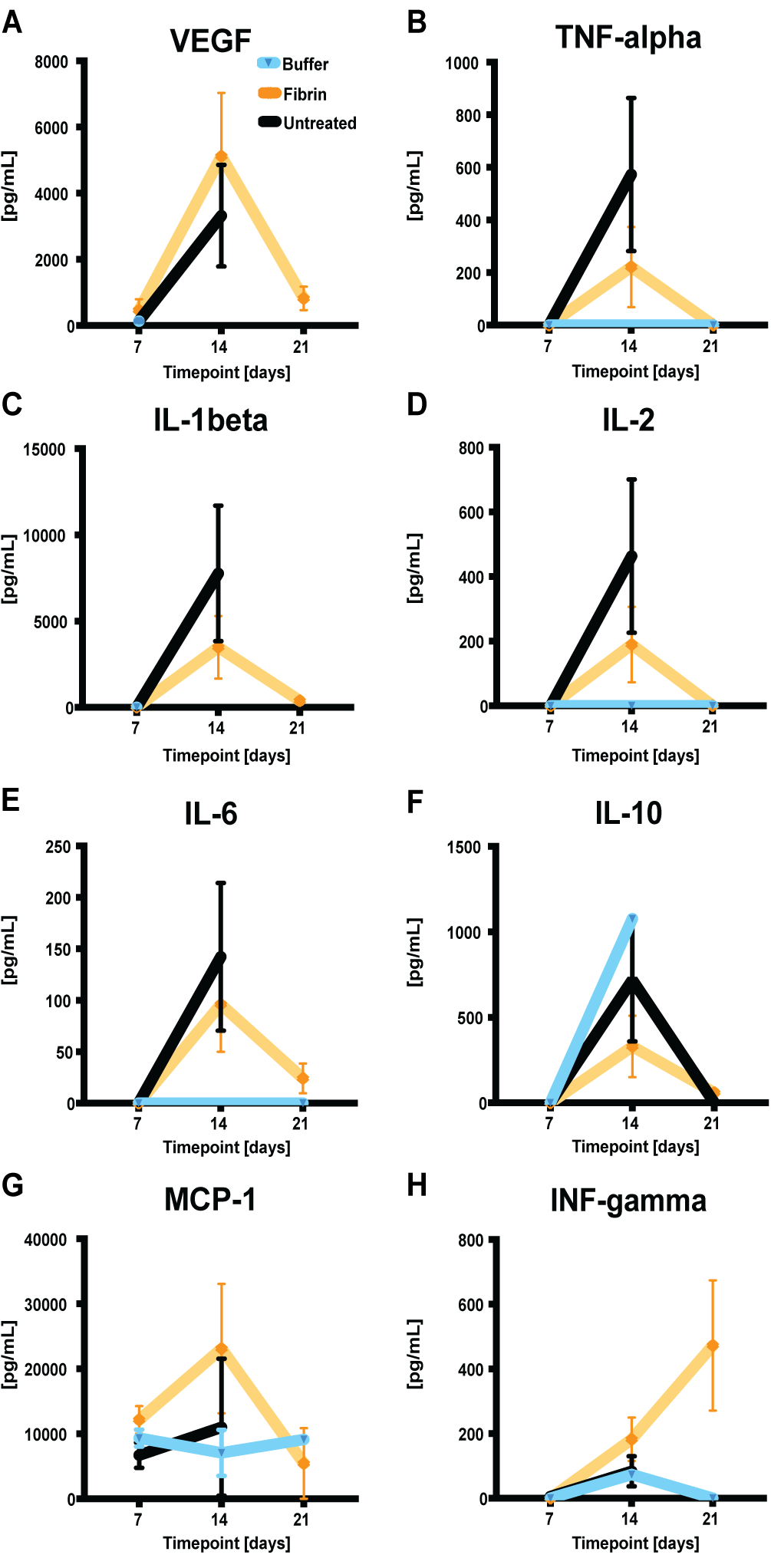

Supplement: Supplementary file 4 [file Image2.tif]

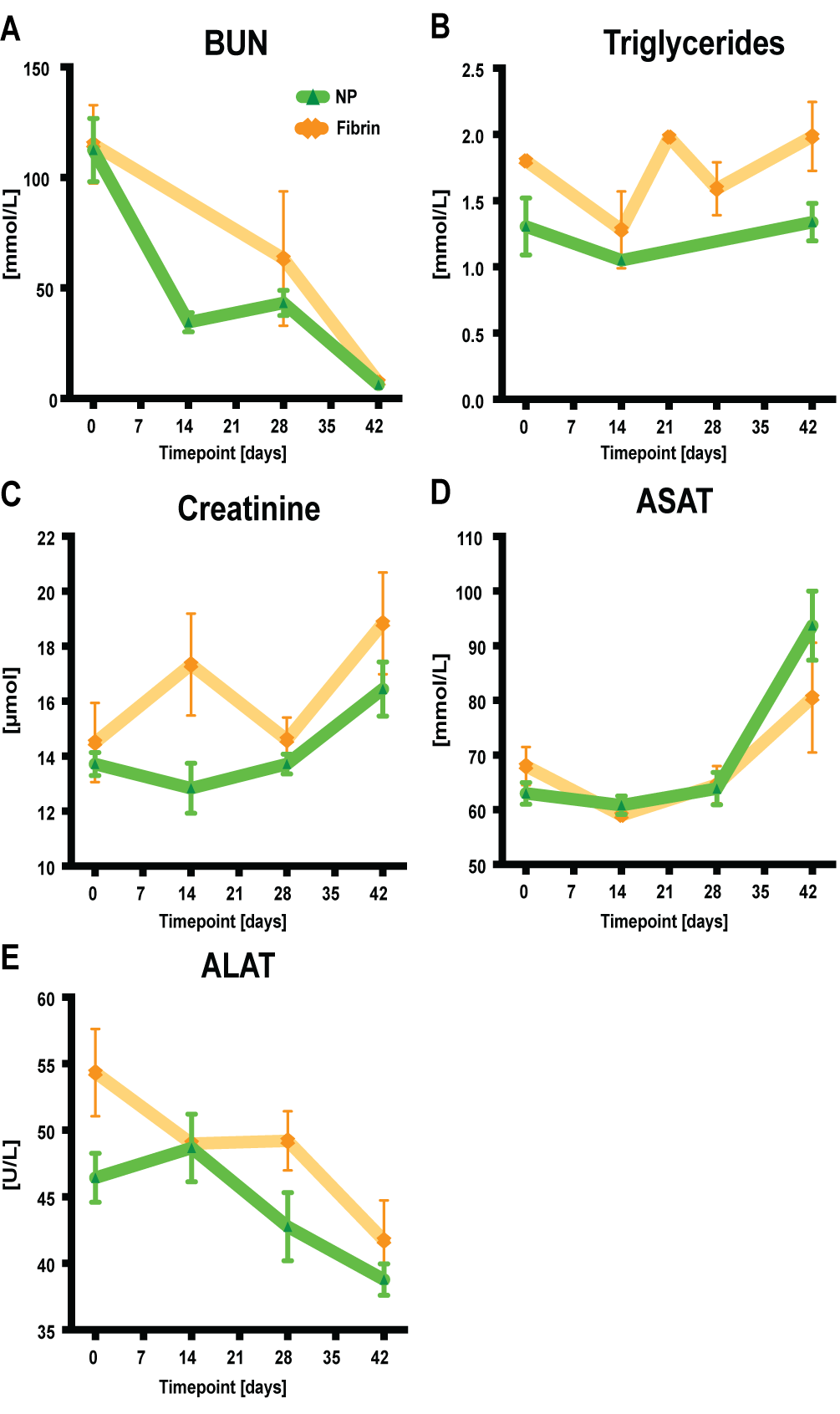

Supplement: Supplementary file 5 [file Image1.tif]
